# Elevated C-reactive protein levels and *ARMS2/HTRA1* gene variants in subjects without age-related macular degeneration

**Published:** 2010-12-31

**Authors:** Tetsuhiro R. Yasuma, Makoto Nakamura, Koji M. Nishiguchi, Masato Kikuchi, Hiroki Kaneko, Toshimitsu Niwa, Nobuyuki Hamajima, Hiroko Terasaki

**Affiliations:** 1 Department of Ophthalmology, Nagoya University Graduate School of Medicine, Nagoya, Japan; 2 Department of Advanced Medicine for Uremia, Nagoya University Graduate School of Medicine, Nagoya, Japan; 3 Department of Preventive Medicine/Biostatistics and Medical Decision Making, Nagoya University Graduate School of Medicine, Nagoya, Japan

## Abstract

**Purpose:**

To investigate the association between the serum high sensitivity C-reactive protein (hs-CRP) levels and variants in age-related maculopathy susceptibility 2 (*ARMS2*)/HtrA serine peptidase 1 (*HTRA1*) genes in normal subjects with no evidence of age-related macular degeneration (AMD).

**Methods:**

After clinical evaluation, information related to medical and social history was collected from 476 Japanese individuals (age range 17–89 years) along with blood samples. These subjects were medical checkup participants recruited at Nagoya University Hospital with no macular disease, as confirmed by fundus photographs. Serum hs-CRP levels were measured using a highly sensitive latex aggregation immunoassay. The genotypes of three polymorphisms in the *ARMS2/HTRA1* locus, i.e., *372_815del443ins54 (del/ins), rs10490924, and rs11200638 were determined using direct sequencing and/or PCR-based assays. After the haplotype was constructed and analyzed, the associations between hs-CRP levels and representative del/ins genotypes were studied with and without adjustment for potential confounding factors.

**Results:**

All three polymorphisms in the *ARMS2/HTRA1* region were in almost complete linkage disequilibrium. Haplotype analyses showed the existence of only two common haplotypes, together comprising 98.9%. Regression analyses showed that the level of hs-CRP was positively correlated with increasing age. This age-dependent increase of hs-CRP levels was greatest in those with homozygous del/ins alleles and lowest in those with homozygous wild-type alleles, which was significant assuming an additive model for gene-dosage association (univariate analyses: p=0.016, multivariate analyses including smoking status, past medical history, and BMI: p=0.043). Consequently, the level of hs-CRP was greatest in those with homozygous del/ins alleles and lowest in those with homozygous wild-type alleles when subjects older than 60 were analyzed. This was significant assuming an additive model for gene-dosage association (univariate analyses: p=0.032).

**Conclusions:**

An age-dependent elevation of serum hs-CRP levels may be accelerated in normal subjects with one or two risk alleles in the *ARMS2/HTRA1* locus compared to those with homozygous wild-type alleles. The results of the current study show that the as-yet undetermined function of variants in the *ARMS2/HTRA1* locus might be linked to inflammation, possibly contributing to the development of neovascular AMD.

## Introduction

Advanced age-related macular degeneration (AMD) is a major cause of legal blindness in elderly people in industrialized countries [1-3]. In Asian populations, including Japanese people, most vision-threatening cases of AMD are of the neovascular type, although central geographic atrophy is less frequent [4,5]. The cause of AMD is complicated because multiple genetic and environmental factors are involved in its pathogenesis. Epidemiological studies have indicated that factors such as age, smoking, gender, obesity, hypertension, and genetic background are associated with AMD [4,6]. Recent progress in research on AMD has provided increasing evidence that inflammatory processes [3,7-10] and oxidative stress [3,10,11] contribute to the pathogenesis of AMD. One well documented alteration of inflammatory markers in AMD patients is the elevation of the serum C-reactive protein (CRP) level [4,12-15]. These levels are also associated with disease progression [16].

Among the several disease-associated genetic variations reported to date [17], those in the 10q26 area are the major genetic risk factors for AMD in Asian people [18-20]. Meanwhile, a polymorphism in another genetic locus (1q32, Y402H variant in the complement factor H [*CFH*] gene) strongly influences the pathology in Caucasian people [21-23]. Currently, two genes residing in the 10q26 area- age-related maculopathy susceptibility 2 (*ARMS2*), also called *LOC387715*, and HtrA serine peptidase 1 (*HTRA1*) are potential candidates to be classified as the AMD-susceptibility gene [18,20]. The risk variants in the *ARMS2/HTRA1* genes include *372_815del443ins54 (del/ins) polymorphism [24] and rs10490924 and rs11200638 single nucleotide polymorphisms (SNPs) [20]. The del443ins54 polymorphism, residing within the polyadenylate region of the *ARMS2* gene, might reduce ARMS2 protein expression [24] and might paradoxically increase the level of *HTRA1* gene products through unknown mechanisms [25]. To date, no analysis of the biologic consequence of the SNP rs10490924 causing a missense alteration (Ala69Ser) in the *ARMS2* gene has been reported. The function of rs11200638, located in the promoter region of the *HTRA1* gene, is controversial; reports have described both increased and unchanged transcription of the gene as a result of the variant [25-27]. Moreover, these three variants are mutually indistinguishable using genotype-phenotype association analyses because they are in almost complete linkage disequilibrium, all residing within approximately 6 kb [24]. Therefore, the mechanisms underlying the contributions of these variants to the pathogenesis of AMD remain unclear.

This study examined the relationship between *ARMS2/HTRA1* gene variants and serum high sensitivity CRP (hs-CRP) levels in normal individuals to assess the biologic effects of the risk allele.

## Methods

### Study subjects

The research protocol was designed in compliance with the Declaration of Helsinki and approved by the institutional review board of the Nagoya University School of Medicine. Written informed consent for providing medical information and blood samples was obtained from each participant. All subjects were ethnic Japanese, residents of the same area of Japan (Chubu, central Japan), and were enrolled in disease prevention programs at Nagoya University Hospital. They were all reportedly unrelated. Most subjects included in this study were described in a previous report [4].

Among the examinees, only subjects with no evidence of even the early stages of AMD, including drusen formation, were selected after evaluation of the fundus photographs by ophthalmologists. Consequently, 476 subjects with no macular degeneration (291 men, 185 women) were recruited [4,28].

### Health and lifestyle factors

All participants completed a health and lifestyle questionnaire, including questions related to smoking and alcohol consumption. The height and weight of each patient was recorded and body mass index (BMI) was calculated as mass (kg) divided by height squared (m^2^).

### CRP Analysis

Serum hs-CRP levels were measured using a highly sensitive latex aggregation immunoassay (Nanopia CRP; Daiichi Pure Chemicals Co. Ltd., Tokyo, Japan) with an analyzer (Hitachi 7170; Hitachi High-Technologies Co., Ltd. Tokyo, Japan). The lower limit of detection was 0.03 mg/l. The high reproducibility of this assay has been reported previously [4].

### Genotyping

The genotypes of *ARMS2/HTRA1* variants were determined using direct sequencing and/or PCR-based assays [4]. In brief, genomic DNAs were extracted from peripheral blood using a kit (QIAamp DNA Blood Maxi; Qiagen Inc., Tokyo, Japan). The DNA fragments, including the variants, were amplified using PCR. To determine the genotypes of rs10490924 and rs11200638 in the *ARMS2/HTRA1* locus, sequences were extracted using a BigDye Terminator v3.1 Cycle Sequencing Kit (Applied Biosystems, Tokyo, Japan) and a genetic analyzer (ABI Prism 3100; Applied Biosystems). The genotypes of the del/ins variants in the *ARMS2/HTRA1* locus were studied based on the size of the PCR-amplified DNA fragments visualized in agarose gel electrophoresis and/or by direct sequencing, as described above.

### Statistical analysis

The indexes of linkage disequilibrium (D’) among three *ARMS2/HTRA1* polymorphisms were calculated. The expectation-maximization algorithm was applied to estimate and analyze the haplotypes of these polymorphisms. Analyses to compare the demographic characteristics among the three different del/ins genotypes, representing three combinations of two different major haplotypes, were performed either using the Kruskal–Wallis test (age average), median test (age median), exact test (gender, medical history, and smoking and drinking habits), or analysis of variance (ANOVA; BMI). Hardy–Weinberg expectations were tested using exact tests [29]. Linear regression analysis was performed to examine the association between the hs-CRP levels and age. For multiple regression analyses applied to control for the demographic characteristics, an additive (but not dominant or recessive) gene-dosage model was assumed, as suggested previously [18]. Furthermore, linear regression analysis was performed to estimate the effect of haplotype on hs-CRP, assuming an additive, dominant, and recessive gene-dosage model. Differences in hs-CRP levels on a natural logarithmic scale (showing a normal distribution) between genotypes were examined using *t*-tests and ANOVA.

For the calculations, PASW Statistics 18 (SPSS Inc., IBM Japan, Ltd., Tokyo, Japan) and *R* (The *R* Foundation for Statistical Computing; genetics and haplo.stats library) [30] were used; p<0.05 was considered significant.

## Results

### Distributions of *ARMS2/HTRA1* genotypes

The three polymorphisms analyzed in the current study––del/ins polymorphism, SNP rs10490924, and SNP rs11200638-were in almost complete mutual linkage disequilibrium (D’>0.9997), which was further supported by haplotype analyses showing the existence of only two common haplotypes in the relevant genomic region, together comprising 98.9% (Table 1). This assures there is little difference in overall interpretation of the present data for all three polymorphisms. Therefore, we indiscriminately chose and analyzed del/ins polymorphisms to represent the genotypes of *ARMS/HTRA1* loci. To avoid confusion, homozygotes with two wild-type alleles, heterozygotes with one del/ins allele, and homozygotes with two del/ins alleles are designated respectively as wild-types, heterozygotes, and homozygotes. In this case, the del/ins allele is the AMD risk allele [24]. The respective frequencies of genotypes for the wild-types, heterozygotes, and homozygotes were 176 (37%), 236 (50%), and 64 (13%), as shown in Table 2. The result of a Hardy-Weinberg equilibrium test showed no marked deviation from the expected distribution of genotypes (p=0.119).

**Table 1:** Estimated haplotype frequencies.

**Category**	rs10490924	**del-ins**	rs11200638	**haplotype frequency**
Haplotype	G (wild-type)	wild-type	G (wild-type)	61.77%
	T (risk)	del-ins	A (risk)	37.19%
	G (wild-type)	del-ins	A (risk)	0.95%
	T (risk)	del-ins	G (wild-type)	0.11%

Haplotype frequencies were estimated using expectation-maximization algorithm, which were presented in the order of frequency (top to bottom).

**Table 2:** Demographic characteristics by *372_815del443ins54 genotypes.

	*372_815del443ins54 genotype				
**Category**	**Wild-type**	**Heterozygote**	**Homozygote**	**Total**	**p value**
number	176 (37.0%)	236 (49.6%)	64 (13.4%)	476	0.11
age average (SD)	49.9 (12.1)	49.0 (13.3)	51.8 (12.6)	49.7 (12.8)	0.29
age median (range)	49 (26–77)	49 (17–89)	53 (25–81)	49 (17–89)	0.36
female gender	65 (36.9%)	88 (37.3%)	32 (50.0%)	185 (38.9%)	0.14
BMI average (SD)	23.0 (3.1)	22.6 (3.1)	22.4 (2.8)	22.7 (3.1)	0.38
**History of**					
hypertension	17 (15.6%)	32 (13.6%)	10 (9.7%)	59 (12.4%)	0.32
hyperlipidemia	22 (12.5%)	32 (13.6%)	13 (20.3%)	67 (14.1%)	0.29
diabetes	7 (4.0%)	9 (3.8%)	3 (4.7%)	19 (4.0%)	0.89
stroke	6 (3.4%)	5 (2.1%)	4 (6.3%)	15 (3.2%)	0.2
cardiovascular disease	2 (1.1%)	9 (3.8%)	2 (3.1%)	13 (2.7%)	0.23
**Smoking habit**					
present	38 (21.6%)	48 (20.3%)	9 (14.1%)	95 (20.0%)	
former	50 (28.4%)	74 (31.4%)	11 (17.2%)	135 (28.4%)	0.061
never	88 (50.0%)	114 (48.3%)	44 (68.8%)	246 (51.7%)	
**Drinking habit**					
once a week or more	88 (50.0%)	132 (55.9%)	23 (35.9%)	243 (51.1%)	
occasional	9 (5.1%)	10 (4.2%)	2 (3.1%)	21 (4.4%)	0.049
no drinking habit	79 (44.9%)	94 (39.8%)	39 (60.9%)	212 (44.5%)	

Demographic characteristics were shown according to *372_815del443ins54 genotypes. P values were calculated to statistically evaluate the differences among three groups. Exact test of Hardy–Weinberg equilibrium (number), Kruskal–Wallis test (age average), median test (age median), exact test (gender, medical history, and smoking and drinking habit), ANOVA (BMI) were applied to calculate P values. Abbreviations: SD, standard deviation; BMI, body mass index

### Demographic characteristics of study subjects

The demographic characteristics of all participants are presented in Table 2. The medical and social backgrounds of the participants were compared among three different genotypes in del/ins locus in normal subjects (mean age of 49.7 years; range 17-89). No significant difference in background characteristics was found between genotypes, except for drinking habits (Table 2). A small difference in the smoking history was not significant (p=0.061).

### Accelerated increase of hs-CRP levels with aging in subjects with del/ins variants

**Figure 1:**
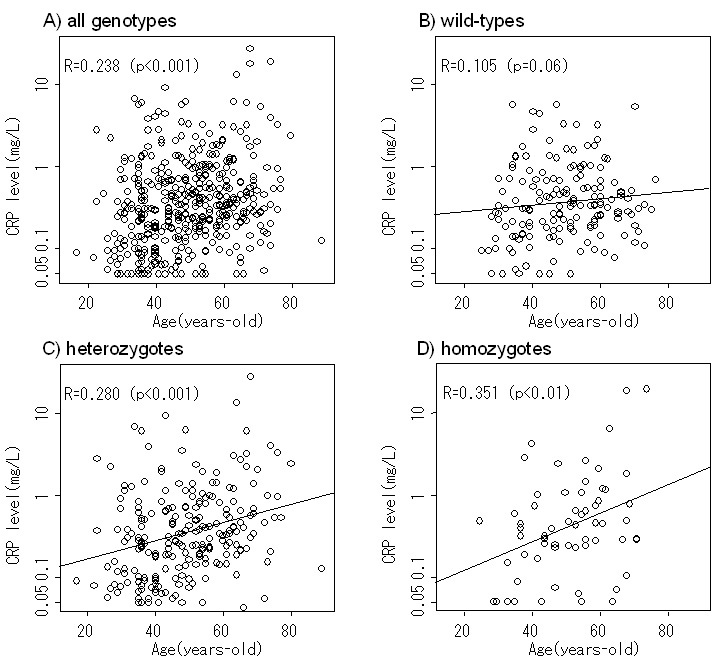
Scatter plots of ages and serum hs-CRP levels (mg/l). Panel **A** was derived from all subjects comprising three genotypes. Panels **B**-**D**, respectively show data from wild-types, heterozygotes, and homozygotes. The linear regression equations are shown as solid, straight lines. Abbreviations: CRP represents C-reactive protein.

The relationship between the measured hs-CRP levels and age is displayed in scatter plots (Figure 1A) and in Table 3, which revealed an age-dependent increase in the levels. The regression coefficients were 0.022 (*R*=0.238, p<0.001; Table 3). When the association was analyzed separately for three different del/ins genotypes (Figure 1B-D), the hs-CRP levels were correlated significantly with age in heterozygotes and homozygotes. Correlation was weak and non-significant among wild-types. The respective regression coefficients were 0.009 (*R*=0.105, p=0.06), 0.026 (*R*=0.280, p<0.001), and 0.040 (*R*=0.351, p=0.004) for wild-types, heterozygotes, and homozygotes. These results showing higher coefficients in those with AMD risk variants were significant (p=0.016), indicating that the increase in hs-CRP levels with aging was accelerated in those with del/ins alleles compared to those without. Next, we examined whether the conclusions hold true after adjustment for demographic characteristics. Analyses of the effects of demographic characteristics (age, gender, smoking habits, alcohol consumption, BMI, and past medical history) and del/ins genotypes on serum hs-CRP levels revealed interaction of age and del/ins genotypes (two-factor interaction; p=0.043) as an independent influential factor. Additionally, BMI (p<0.001) and a history of stroke (p=0.012) were shown to affect the hs-CRP levels. The overall explanatory power of the multivariate analyses was 0.183 (meaning that 18.3% of variation in the hs-CRP levels could be explained by all factors, including genotypes, ages, and other demographic characteristics, analyzed in the multivariate analyses).

**Table 3:** Regression analyses of hs-CRP levels.

**Genotype**			
**372_815del443ins54 genotype**		**p value †**	**p value ‡**
Wild-type logarithm of serum CRP=	0.0097x [Age]-3.7	0.063	
Heterozygote	0.025x [Age]-4.6	2.4×10^−7^	0.016
Homozygote	0.039 x [Age]-5.2	0.0041	
Total	0.022x [Age]-4.4	2.9×10^−7^	
**Effect of risk haplotype**			
**gene-dosage model**	**estimated regression coefficients (age)**		**p value**
Dominant	wild-type	0.0098	0.054
	heterozygote + homozygote	0.027	
Recessive	wild-type + heterozygote	0.02	0.15
	homozygote	0.04	
Additive	wild-type	0.0097	0.027
	heterozygote	0.025	
	homozygote	0.04	

**†**The regression coefficients for age were statistically significant in heterozygotes (p<0.001), homozygotes (p=0.004), and all genotypes combined (p<0.001). **‡**The regression coefficients for age were significantly different among three genotypes (p=0.016)

We further assessed the association of age and hs-CRP between haplotypes and assessed the difference in the regression coefficients using two-tailed analyses (Table 3). Analyses of haplotypes required an assumption about the mode of influence (dominant, recessive, or additive model). Therefore, analyses identical to those for genotypes were impossible. Results showed that the risk haplotype had a significantly larger regression coefficient assuming an additive model (p=0.027), but not a dominant (p=0.054) or a recessive model (p=0.15).

### Elevated Serum hs-CRP levels in subjects with del/ins variant

Among all subjects, geometric means of the serum hs-CRP levels were, respectively, 0.37, 0.35, and 0.41 mg/l for wild-types, heterozygotes, and homozygotes. These differences in hs-CRP levels were not significant (p=0.82; Table 4). However, when subjects old enough to develop AMD were selected (more than 60 years old) and examined, means of the serum hs-CRP levels were, respectively, 0.35, 0.60, and 0.71 mg/l for wild-types, heterozygotes, and homozygotes. These differences in hs-CRP levels were significant (p=0.032; Table 4). Meanwhile, when the means of hs-CRP levels were compared between younger and older groups within the same genotype, levels were significantly lower in younger subjects than in those over 60 years old in the heterozygotes group (p<0.001, 0.30 versus 0.60 mg/l), but not in the homozygotes group (p=0.15, 0.34 versus 0.71 mg/l) or the wild-types group (p=0.69, 0.38 versus 0.35 mg/l).

**Table 4:** Serum hs-CRP levels by *372_815del443ins54 genotypes.

**All ages**	**Number**	**Geometric mean**	**GSD**
wild-type	166 (36.6%)	0.372	2.836
heterozygote	226 (49.9%)	0.349	3.379
homozygote	61 (13.5%)	0.409	4.002
total	453	0.365	3.255
**Over 60 years old**	**Number**	**Geometric mean**	**GSD**
wild-type	36 (35.0%)	0.353	2.261
heterozygote	52 (50.4%)	0.602	3.601
homozygote	15 (4.3%)	0.713	6.19
total	103	0.512	3.52
**p values for the difference in geometric means**	**All ages**	**Over 60 years old**	
dominant model	0.79	0.011	
recessive model	0.41	0.43	
additive model	0.81	0.032	

Serum hs-CRP levels on subjects of all ages, over 60 years-old were shown. p values for the difference in geometric means among *372_815del443ins54 genotypes assuming dominant, recessive, or additive gene-dosage model were shown. Abbreviations: CRP, C-reactive protein;GSD, geometric standard deviation.

## Discussion

In this study, the results suggest the association of the AMD risk alleles in the *ARMS2/HTRA1* gene and an increased rate of age-dependent elevation in the hs-CRP level in normal subjects without macular disease. Consequently, when participants over 60 years of age were selected and examined, the hs-CRP levels were elevated in those carrying one or more copies of the risk allele. Meanwhile, the mechanisms underlying the age-dependent alteration of hs-CRP remains unknown.

Despite the growing consensus that chronic inflammation is an important factor in the pathogenesis of AMD, the association of *ARMS2/HTRA1* and systemic inflammatory markers, including CRP, has not been reported. In this study, we produced evidence supporting the notion that the *ARMS2/HTRA1* risk allele for AMD is linked directly or indirectly to chronic systemic inflammation. Because the elevated CRP is also associated with increased progression of AMD [16], these findings suggest that the risk allele might contribute to the development and progression of AMD. Meanwhile, the results of the current study did not include the genotype analyses of the *CRP* gene in chromosome 1q21-q23, which might also influence the level of CRP [31]. However, no association between the *CRP* gene and AMD has been reported [32,33].

Another AMD risk variant, Y402H in *CFH*, is reportedly uncommon among Japanese [34]. Nevertheless, we analyzed the correlation between the serum hs-CRP levels and *CFH* Y402H genotypes (data not shown). The allele frequencies of Y402H variants were approximately 5% in our study population, and no measurable difference was found in the geometric means of hs-CRP levels between genotypes, possibly because of the small number of subjects with the Y402H allele. Notably, a previous report found no association between the Y402H allele and CRP levels in a Caucasian cohort [35].

To date, the functions of *ARMS2* and *HTRA1* genes remain largely unknown. The ARMS2 protein is reportedly expressed in the retina [26], specifically in the mitochondria-rich ellipsoid region of the photoreceptors [24]. Together with results showing co-localization of the protein and mitochondria in cells transfected with the *ARMS2* gene, the functional role of ARMS2 in mitochondrial homeostasis was proposed [24,26]. However, Arg38X polymorphism (rs2736911), presumably resulting in the lack of this gene product, is frequently found in the non-risk allele of the *ARMS2* gene [19,25], which indicates that the loss of function of this gene is less likely to contribute to AMD pathogenesis [25]. Meanwhile, the role of HTRA1 protein is also incompletely characterized, aside from observations showing its expression in drusen from human eyes [27] and the inhibitory role in signaling by transforming growth factor (TGF)-β family proteins [36,37]. Down-regulation of TGF-β signaling might induce aberrant invasion of choroidal vasculatures by promoting proliferation, tube formation, and migration of vascular endothelial cells [27,38]. Nevertheless, the current biologic data for these genes are not sufficient enough to specify which gene or combination of genes is associated with the inflammation that confers susceptibility for AMD. At the same time, the presence of another uncharacterized variation/gene at this locus responsible for the elevation of CRP and systemic inflammation cannot be ruled out.

In conclusion, normal subjects with AMD risk alleles in the *ARMS2/HTRA1* locus may be at risk of increased hs-CRP levels and chronic systemic inflammation with aging, which probably heightens the risk of developing AMD. The results of this study provide important insight into the role of gene variants in the *ARMS2/HTRA1* locus, the major genetic risk for the development of neovascular AMD in Asian people.
